# Association between Statin Use and Short-Term Outcome Based on Severity of Ischemic Stroke: A Cohort Study

**DOI:** 10.1371/journal.pone.0084389

**Published:** 2014-01-21

**Authors:** Bo Song, Yilong Wang, Xingquan Zhao, Liping Liu, Chunxue Wang, Anxin Wang, Wanliang Du, Yongjun Wang

**Affiliations:** Department of Neurology, Beijing Tiantan Hospital, Capital Medical University, Beijing, China; INSERM, Paris, France

## Abstract

**Background:**

Statins reportedly improve clinical outcomes for ischemic stroke patients. However, it is unclear whether the contribution of statin treatment varies depending on the severity of stroke. We sought to investigate the relationship between statin use and the outcome of acute first-ever ischemic stroke patients stratified by stroke severity.

**Methods:**

A total of 7,455 acute first-ever ischemic stroke patients without statin treatment before onset were eligible from the China National Stroke Registry. A National Institutes of Health Stroke Scale (NIHSS) score of 0 to 4 was defined as minor stroke, and a NIHSS score of >4 was defined as non-minor stroke. We analyzed the association between statin use during hospitalization and mortality as well as functional outcome (measured by a modified Rankin Scale score of 0–5) at 3 months after onset using multivariable logistic regression models.

**Results:**

A total of 3,231 (43.3%) patients received statin treatment during hospitalization. Multivariable analysis showed that statin use during hospitalization decreased mortality of ischemic stroke patients (OR, 0.51; 95%CI, 0.38–0.67), but did not improve poor functional outcomes (OR, 0.95; 95CI%, 0.81–1.11) at 3 months. The interaction between statin use and stroke severity was significant both in dependence and death outcome (P = 0.04 for dependence outcome, P = 0.03 for death outcome). After stratification by stroke severity, statin use during hospitalization decreased the mortality of stroke (OR, 0.44; 95%CI, 0.31–0.62) and poor functional outcome (OR, 0.73; 95%CI, 0.57–0.92) at 3 months in the non-minor stroke group.

**Conclusions:**

Statin use during hospitalization may improve the clinical outcome of acute first-ever ischemic stroke depending on the severity of stroke. Non-minor stroke patients may obtain benefit from statin treatment with improvements in poor functional outcomes and mortality.

## Introduction

Statins are inhibitors of 3-hydroxy-3-methylglutaryl coenzyme reductase and have potential pleiotropic effects on stroke in addition to their lipid-lowering properties. Many observational studies have revealed that statin use before or after stroke onset can decrease mortality[1]–[3] and improve short-term[4]–[6] and long-term[7]–[8] outcomes of ischemic stroke. Statin use during hospitalization on ischemic stroke and transient ischemic attack (TIA) patients can decrease the recurrence of composite outcomes including stroke, ischemic heart disease, and all-cause death.[9] In addition, discontinuation of statins after ischemic stroke may lead to unfavorable outcomes.[10]

Researches revealed that the clinical outcome of ischemic stroke patients depended on stroke severity, and minor stroke patients had a better prognosis.[11]–[13] There was only one study focused on clinical outcome of statin treatment to the minor stroke or TIA. This randomized controlled study, Fast assessment of stroke and transient ischemic attack to prevent early recurrence (FASTER), including 396 individuals, showed that simvastatin use did not affect mortality or functional outcomes of minor stroke and TIA patients.[14] Therefore, it is worthwhile to speculate whether statin treatment has any clinical effect on the outcome of ischemic stroke or whether the association between statin treatment and outcome varies depending on stroke severity. There have been no reports involving a Chinese population on this topic. In the present study, we assessed the association between statin therapy during hospitalization and the outcome of acute first-ever ischemic stroke based on the data of the China National Stroke Registry (CNSR).[15]

## Subjects and Methods

### Data source

The CNSR is a nationwide, prospective, hospital-based registry aimed at evaluating risk factors, clinical characteristics, treatment, prevention status survey, and prognosis of acute stroke from September 2007 to August 2008 in China. Detailed information of the design of the CNSR registry has been previously published.[15]–[16]

Trained physicians recorded all information of enrolled stroke patients, including demography, vascular risk factors, clinical manifestations, the National Institutes of Health Stroke Scale (NIHSS) score,[17] laboratory examinations, clinical diagnosis, treatment, secondary prevention, hospitalization time, and outcome. Follow-up by trained research personnel at Beijing Tiantan Hospital was carried out by telephone interview.

### Ethics Statement

This research was approved by the central Institutional Review Board at Beijing Tiantan Hospital. All patients or their legally authorized representatives signed an informed consent form.

### Study population

Ischemic stroke was diagnosed based on World Health Organization criteria[18] with brain CT or MRI evidence. The inclusion criteria were: (1) first-ever ischemic stroke onset within 14 days; (2) no statin use before stroke onset. We excluded patients diagnosed with TIA (The TIA diagnosis in the present study was based on WHO TIA diagnostic criteria,[18] which defines a TIA as an acute loss of focal cerebral or ocular dysfunction lasting less than 24 h attributed to embolic or thrombotic vascular diseases), hemorrhagic stroke, and those with unclear clinical information or a life expectancy of <1 year because of severe disorders such as cancer and hepatic disease. Patients were divided into statin use group and non-statin use group according to statin use record during hospitalization in this cohort study.

Detailed baseline data were registered prospectively using paper case report forms (CRF) designed specifically for this study, including age, sex, severity of stroke, current or previous smoking, moderate or heavy alcohol consumption (≥2 standard volume of alcohol consumption per day), and so on. The severity of stroke was evaluated by the NIHSS within 24 hours after admission. A NIHSS score of 0 to 4 was defined as minor stroke, [19] and a NIHSS score >4 was defined as non-minor stroke. Patients who took statins regularly by prescription before discharge were assigned to the statin use group irrespective of type or dosage. Information on demography and vascular risk factors including history of hypertension, diabetes mellitus(DM), coronary heart disease (CHD), atrial fibrillation(AF) and TIA was obtained from patients' self-reports with medical records or treatment data. Medications during hospitalization included use of antithrombotic, antihypertensive, antidiabetic drugs and statins. Antithrombotic medication included antiplatelet and anticoagulation drugs used during hospitalization; thrombolytic treatment was excluded because very few patients took thrombolytic agents; Antihypertensive medication included any treatment of venous or oral antihypertensive drugs. Antidiabetic medication included insulin and oral hypoglycemic drugs. Stroke outcome was assessed from mortality (due to any causes) rates and the modified Rankin Scale (mRS) during the follow-up period. For the purpose of this study, patients with a mRS score of >2 were defined as having dependency, which had a poor functional outcome.

### Statistical analysis

All data were analyzed by SAS version 9.1.3 statistical software. Demographic data and clinical manifestations in ischemic stroke individuals with statin therapy during hospitalization were compared with those without statin therapy during hospitalization, and categorical and continuous variables were compared by the χ^2^ and t test, respectively. The associations between statin therapy during hospitalization and death/dependency were analyzed in multivariate binary logistic regression models after adjusting for potential confounders including age, sex, NIHSS score at admission, vascular risk factors such as current or previous smoking, moderate or heavy alcohol consumption and history of hypertension, DM, CHD, AF and TIA; and medication before discharge including antithrombotic treatment, antihypertension treatment, and antidiabetic treatment. Sub-group analysis was performed to explore whether the benefit from statin therapy varied depending on stroke severity. Before that, statistical interaction analysis was deployed to identify the interaction effect between statin use and stroke severity.

## Results

### Patient flow

**Figure 1:**
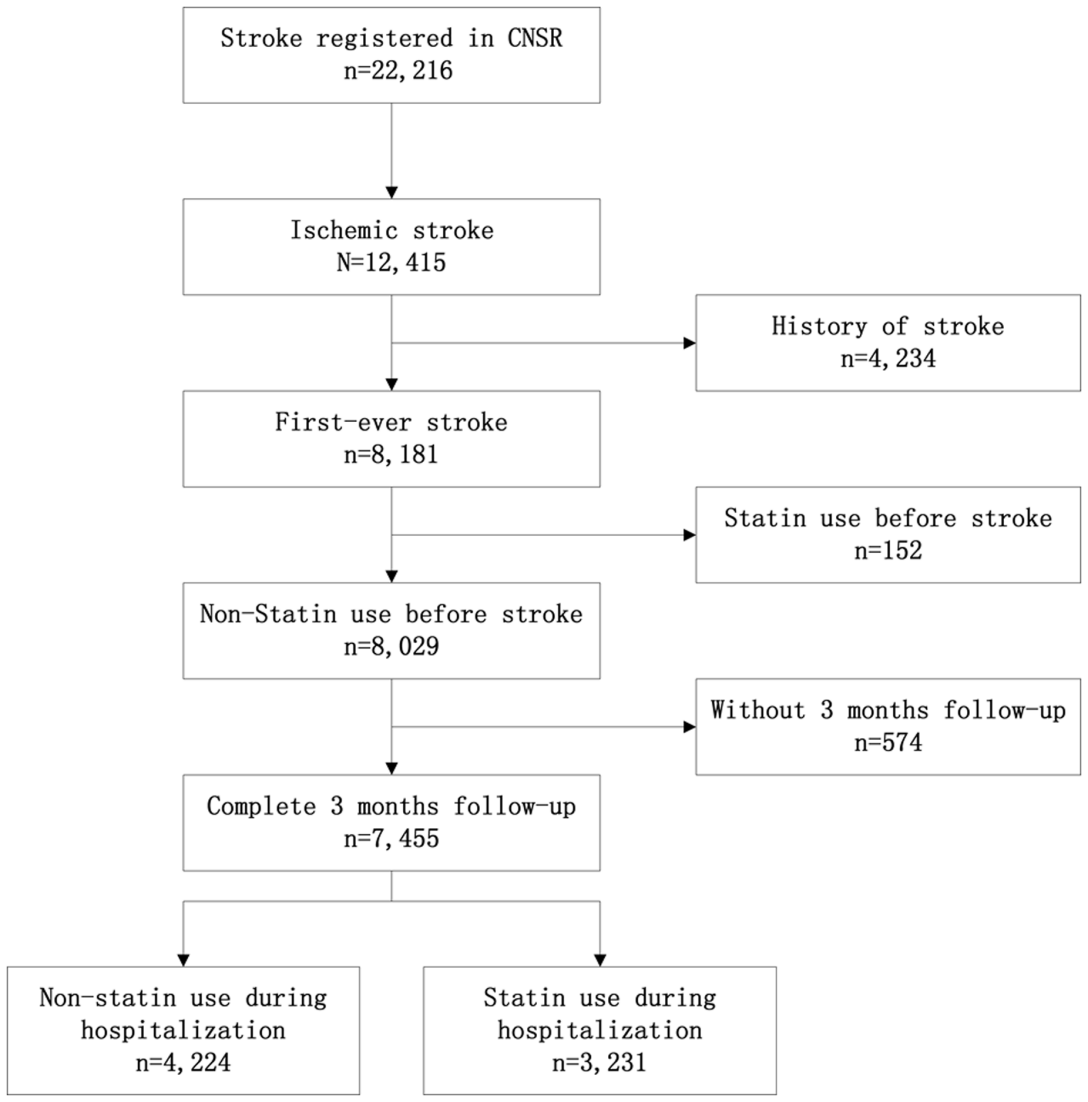
Flow diagram defining the potentially eligible patients with acute stroke for statins treatment.

A total of 22,216 hospitalized acute stroke patients within 14 days after onset were recruited from September 2007 to August 2008, and 12,415 of them were diagnosed with acute ischemic stroke. 8,181(65.9%) patients were first-ever stroke, 152 patients were excluded because of history of statin use before stroke. There were 7,455 eligible patients enrolled after excluding 574(7.2%) patients lost at 3-month follow-up in this study (**Figure 1**).

### Baseline characteristics

The average age of the patients was 64.37±12.80 years old, and 38.5% of them were female. A total of 3,231(43.3%) patients received statin therapy during hospitalization. The baseline demographic information, vascular risk factors, and clinical characteristics are listed in Table 1. The serum levels of cholesterol, triglycerides, low-density lipoprotein were greater in the patients receiving statin therapy during hospitalization. The statin use group also was more likely to prescribed concomitant hypertension, diabetes mellitus, coronary heart disease and atrial fibrillation. More patients in the statin use group received antithrombotic, antihypertensive, and antidiabetic medications. There were more minor stroke patients in the statin use group than the non-statin use group.

**Table 1:** Baseline characteristics of patients according to statin use.

Variable	Non-statin(n = 4224)	Statin(n = 3231)	p-value
Age, mean ± SD, years	64.52±13.17	64.18±12.31	0.11
Gender (Female), n (%)	1639(38.8%)	1232(38.1%)	0.56
Time of onset to admission	15.72±8.32	16.47±8.19	0.13
History of DM, n (%)	692(16.4%)	646(20%)	<0.001
History of hypertension, n (%)	2300(54.5%)	1961(60.7%)	<0.001
History of CHD, n (%)	540(12.8%)	358(11.1%)	0.025
History of AF, n (%)	333(7.9%)	156(4.8%)	<0.001
History of TIA, n (%)	101(2.4%)	82(2.5%)	0.68
smoking, n (%)	1651(39.1%)	1304(40.4%)	0.27
alcohol, n (%)	431(10.2%)	379(11.7%)	0.036
NIHSS, median (IQR)	5(2, 10)	4(2, 8)	<0.001
Minor stroke, n (%)	2111(50.0%)	1768(54.7%)	<0.001
HDL-C, mean ± SD	1.24±0.46	1.22±0.53	<0.001
LDL-C, mean ± SD	2.70±0.85	3.05±1.04	<0.001
TC, mean ± SD	4.60±1.28	5.04±5.01	<0.001
TG, mean ± SD	1.64±1.19	1.92±1.41	<0.001
Medications in-hospital			
Antithrombotic, n (%)	3452(81.7%)	3061(94.7%)	<0.001
Antihypertension, n (%)	1567(37.1%)	1553(48.1%)	<0.001
Antidiabetic, n (%)	739(17.5%)	808(25%)	<0.001
Hospitalization time(days)	16.05±11.83	17.21±10.98	<0.001
Average incomes<1000RMB	1685(46.16%)	1344(43.59%)	<0.001
1000RMB≤Average incomes≤3000RMB	1593 (43.64%)	1392 (45.07%)	
Average incomes >3000RMB	372(10.19%)	347(11.23%)	
Medical insurance	3151(74.6%)	2442(75.58%)	0.33

DM: diabetes mellitus; CHD: coronary arterial disease; AF: atrial fibrillation; Smoking: current or previous smoking; Alcohol: moderate or heavy alcohol consumption; NIHSS: the National Institutes of Health Stroke Scale evaluated within 24 hours after admission; IQR : indicates interquartile range; Minor stroke: NIHSS scale <5; HDL-C: high-density lipoprotein cholesterol; LDL-C: low-density lipoprotein cholesterol; TC: total cholesterol; TG: triglycerides; RMB: Ren Min Bi.

### Outcome of patients

**Figure 2:**
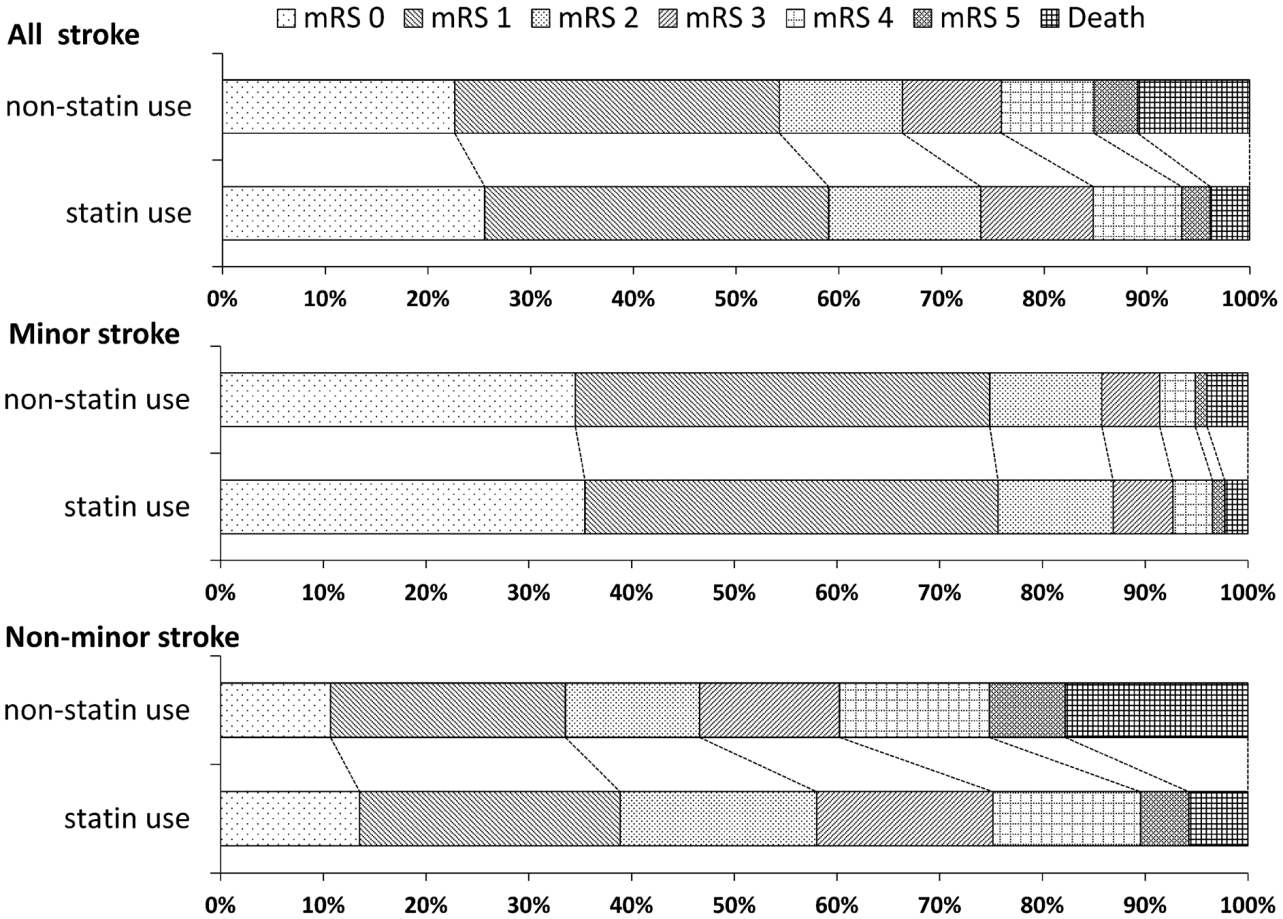
Percentage of mRS scores and death at 3 months for ischemic stroke patients stratified by statin use within hospitalization.

A total of 584 (7.8%) patients died and 1690 (22.7%) became dependent during 3-month follow-up period. In the statin use group, 3.8% patients died, compared with 10.9% patients in the non-statin group (P<0.001). Mortality in the statin use group, whatever minor stroke or non-minor stroke, was lower than the non-statin use group. A total of 22.3% patients in the statin use group were dependent compared with 22.9% patients in the non-statin use group. Less non-minor stroke patients were dependent in the statin use group, but there was no difference in minor stroke patients between statin use group and non-statin group (Table 2). The percentages of mRS scores and death in the two groups were shown in Figure 2.

**Table 2:** Patient Functional Outcomes According to Statin Use at 3 Months.

Variable	Non-statin	Statin	p-value
**All patients**			
Dependence	968(22.9%)	722(22.3%)	0.018
Death	460(10.9%)	124(3.8%)	<0.001
**Minor stroke**			
Dependence	216(10.66%)	192(11.11%)	0.66
Death	84(3.98%)	40(2.27%)	0.006
**Non-minor stroke**			
Dependence	752(43.29)	530(38.43)	0.006
Death	376(17.8)	84(5.74)	<0.001

### Prognosis analysis

**Figure 3:**
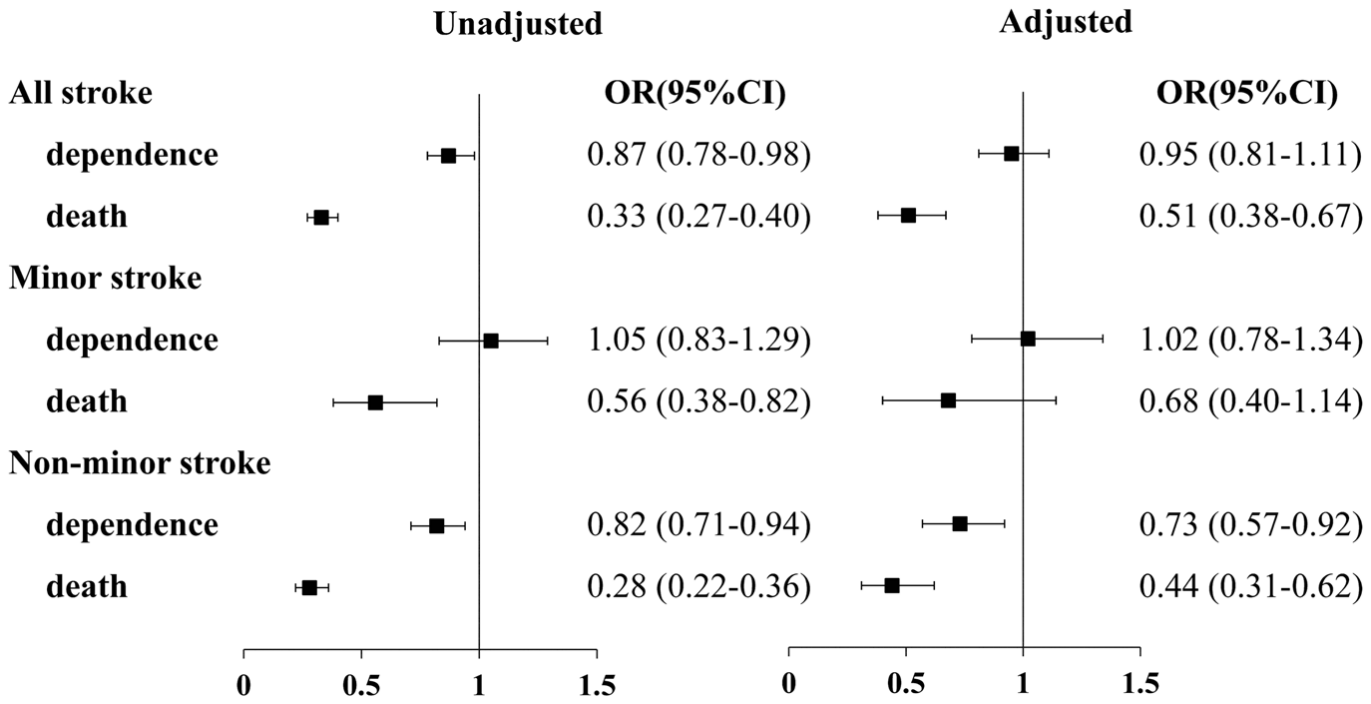
Multivariate analyses of functional outcomes at 3 months for all patients and patients stratified by severity of stroke.

The analyses showed that statin use during hospitalization could decrease mortality at 3 months (OR, 0.33; 95% CI, 0.27–0.40), this effect remained statistically significant after adjusting for confounding variables, such as demographic data, vascular risk factors, stroke severity, and in-hospital medications(OR, 0.51; 95%CI, 0.38–0.67). Statins could improve the functional outcomes of stroke patients at 3 months (OR, 0.87; 95%CI, 0.78–0.98), however, this effect did not reach statistical significant after adjusting for confounding factors (OR, 0.95; 95CI%, 0.81–1.11) (**Figure 3**).

Statistical interaction analysis revealed that there were significant interaction effects between statin use and stroke severity both in dependence and death outcome (P = 0.04 for dependence outcome, P = 0.03 for death outcome). After patients were stratified by stroke severity, non-minor stroke patients obtained a benefit of good functional outcome (OR, 0.73; 95%CI, 0.57–0.92) and decreased mortality from statin therapy during hospitalization (OR, 0.44; 95%CI, 0.31–0.62). However, statin therapy during hospitalization had no impact on mortality (OR, 0.68; 95% CI, 0.4–1.14), and poor functional outcome (OR, 1.02; 95% CI, 0.78–1.34) at 3 months in the minor stroke group (Figure 3). All the factors that could significantly affect the clinical outcome of the patients at 3 months were listed in Table 3.

**Table 3:** Significant Predictors of Clinical Outcome at 3 months.

Variable	ALL stroke OR(95%CI)	Minor stroke OR(95%CI)	Non-minor stroke OR(95%CI)
Dependence			
Age	1.04(1.04–1.05)	1.06(1.04–1.07)	1.04(1.03–1.05)
Gender	1.45(1.2–1.76)	1.41(1.03–1.93)	1.49(1.16–1.9)
History of AF	1.51(1.09–2.51)	1.87(1.1–3.15)	1.4(1.02–1.93)
NIHSS	1.24(1.22–1.26)	1.13(1.03–1.24)	1.21(1.18–1.24)
Antithrombotic	0.69(0.52–0.91)	0.84(0.52–1.35)	0.65(0.46–0.93)
Antidiabetic	1.69(1.41–2.03)	1.97(1.47–2.62)	1.56(1.23–1.97)
Statin	0.95(0.81–1.11)	1.02(0.78–1.34)	0.73(0.57–0.92)
Death			
Age	1.04(1.03–1.05)	1.02(1–1.05)	1.05(1.03–1.06)
Gender	1.21(0.89–1.66)	1.17(0.64–2.14)	1.21(0.84–1.75)
History of AF	1.51(1.06–2.15)	1.77(0.72–4.32)	1.45(1.01–2.25)
NIHSS	1.13(1.11–1.15)	1.06(0.89–1.26)	1.13(1.11–1.15)
Antithrombotic	0.48(0.34–0.69)	0.37(0.19–0.72)	0.52(0.35–0.79)
Statin	0.51(0.38–0.67)	0.68(0.4–1.14)	0.44(0.31–0.62)
Average income<1000RMB	1(Ref)	1	1
Average income >3000RMB	0.68(0.52–0.89)	0.82(0.49–1.37)	0.61(0.44–0.84)
Medical insurance	0.93(0.69–1.25)	0.54(0.31–0.93)	1.14(0.8–1.62)

AF: atrial fibrillation, Smoking: current or previous smoking, NIHSS: the National Institutes of Health Stroke Scale evaluated within 24 hours after admission; RMB:Ren Min Bi.

## Discussion

Our data showed that acute first-ever ischemic stroke patients with a NIHSS score >4 obtained a significant benefit of functional outcome and decreased mortality from statin therapy during hospitalization at 3 months, after adjusting for confounding factors. The results also suggested that moderate and severe ischemic stroke patients but not minor stroke patients could benefit from statin therapy.

Our findings were consistent with other studies about the effect of statins on prognosis of ischemic stroke. Statin use after ischemic stroke onset has been associated with improved functional outcome and decreased post-stroke mortality.[3]–[7] A 1360-case multicenter study found a racial difference in the beneficial effects of statins that Caucasian Americans seemed to benefit more.[4] The results from Taiwan Stroke Registry found that early lipid-lowering therapy during hospitalization could improve composite end point, including all-cause mortality, recurrent stroke, or the occurrence of ischemic heart disease, of the ischemic stroke and TIA patients. [9] In addition, some studies have found that patients of each stroke subtype obtained different benefits from statin therapy; patients with atherothrombosis[20] and/or small vessel infarctions[20]–[21] showed the greatest benefit. Research from Kaiser Permanente Northern California reported that early statin use during stroke hospitalization was strongly associated with improved post-stroke survival, and the patients who received higher doses and earlier treatment in-hospital showed better survival.[3]

Our study also revealed that not all acute ischemic patients would benefit from statin therapy. The reason may be that we analyzed functional outcomes and mortality separately, while other studies considered functional outcomes and mortality to be a compounding endpoint enhancing poor outcomes, which would lead to different results. In addition, after stratified into two groups according to NIHSS scores, minor stroke patients did not obtain benefit from statin therapy, which was consistent with FASTER trial. The reason, we speculate, may be that minor stroke is associated with a better functional outcome and less mortality compared with moderate and severe ischemic stroke. We presume that the effect of statin therapy for minor stroke patients cannot be fully embodied because of their already generally good prognosis, which is unable to be easily further improved.

Biomedical studies have confirmed that statins had a pleiotropic effect via increasing endothelial nitric oxide synthase levels, reducing free radical levels, inhibiting the activity of excitotoxic amino acids and the production of inflammatory mediators, reducing clot formation, enhancing clot dissolution, promoting angiogenesis, and so on.[22] Clinical trials have shown consistent results. An observational trial involving 67 patients with acute ischemic stroke found that simvastatin inhibited increases in the level of serum 8-isoprostane, which was associated with the contribution of oxidative stress to brain ischemia.[23] However, there were some conflicts among several clinical trials in terms of the association between blood inflammatory factors and ischemic stroke in statin therapy. A recent small-sample, randomized controlled trial showed that early-stage statin use after the onset of acute ischemic stroke produced no reduction on the inflammation related biomarkers but actually led to a non-significant increase in mortality and greater proportion of infections in the statin group after 3 months.[24]

To our knowledge, there has not been a large sample, randomized controlled trail which confirmed that statin use in acute ischemic stroke can decrease the mortality and dependence. The FASTER trial showed simvastatin 40 mg daily did not affect mortality or functional outcomes of minor stroke and TIA patients.^[14]^ A meta-analysis made a conclusion that the safety and effectiveness of statin therapy in the early stage of acute ischemic stroke and TIA was unclear based on only a few randomized trials.[25] More large-sample randomized controlled trials are needed to confirm the pleiotropic effect of statins.

As other similar observational studies, there are some limitations in this research. Firstly, it has been controversial whether the dosage of statins impacted on the severity of stroke, [26]–[27]however, we did not collect the category or dosage of statins which made it impossible to evaluate the dose-effect relationship of the benefit of statin. Secondly, the exact time of statin initiation was not analyzed, which made it impossible to identify whether there was a time-dependent effect of statin use. Thirdly, statin discontinuation was not included in this study, while statin withdrawal was associated with increased risk of death or dependence at 90 days.[28] Fourthly, the medications after discharge, which would affect the outcome of ischemic stroke, were not included in the logistic analyses. Finally, as it was an observational study, there were differences between the statin use and non-statin use group in stroke severity, risk factors, personal history, etc. These differences still led to bias although we adjusted for this in the multivariate logistic-regression models.

## Conclusion

Our research shows that statin use during hospitalization could improve the clinical outcome of acute first-ever ischemic stroke depending on the severity of stroke. Non-minor stroke patients would obtain benefit from statin treatment with improvements in poor functional outcomes and mortality. Further prospective studies are needed to confirm our results.
